# Triazido­[tris­(2-pyridyl-κ*N*)methyl­amine]cobalt(III)

**DOI:** 10.1107/S1600536808035162

**Published:** 2008-11-08

**Authors:** Lujiang Hao, Xia Liu

**Affiliations:** a College of Food and Biological Engineering, Shandong Institute of Light Industry, Jinan 250353, People’s Republic of China; b Maize Research Institute, Shandong Academy of Agricultural Science, Jinan 250100, People’s Republic of China

## Abstract

The title compound, [Co(N_3_)_3_(C_16_H_14_N_4_)], was synthesized by hydro­thermal reaction of [Co(NH_3_)_6_](NO_3_)_3_, NaN_3_ and tris­(2-pyrid­yl)methyl­amine. The structure contains two independent complexes in the asymmetric unit, with closely comparable geometry. The Co^III^ atoms are hexa­coordinated by three N atoms from the tridentate tris­(2-pyrid­yl)methyl­amine ligands and three azide ions in a *fac* arrangement. N—H⋯N hydrogen bonds are formed between the amino group and the uncoordinated terminal N atoms of the azide ligands.

## Related literature

For other complexes containing the tris­(2-pyrid­yl)methyl­amine ligand, see: Arnold *et al.* (2001 ▶). For related Co^III^ triazide complexes, see: Ma *et al.* (2000 ▶); Chun & Bernal (2000 ▶).
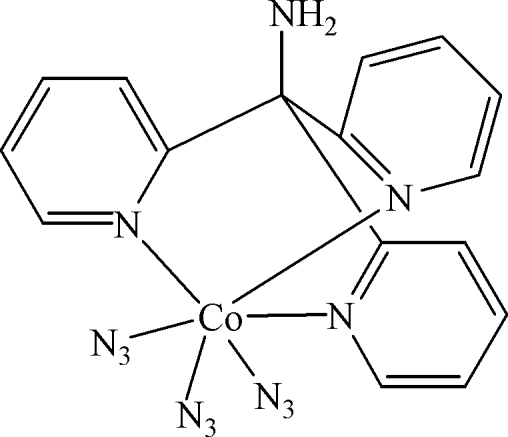


## Experimental

### 

#### Crystal data

[Co(N_3_)_3_(C_16_H_14_N_4_)]
*M* *_r_* = 447.33
Triclinic, 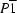

*a* = 10.2063 (3) Å
*b* = 13.6760 (6) Å
*c* = 14.4735 (2) Å
α = 68.150 (3)°
β = 77.114 (2)°
γ = 80.249 (2)°
*V* = 1819.53 (10) Å^3^
*Z* = 4
Mo *K*α radiation
μ = 0.98 mm^−1^
*T* = 295 (2) K
0.20 × 0.14 × 0.12 mm

#### Data collection

Bruker APEXII CCD diffractometer
Absorption correction: multi-scan (*SADABS*; Bruker, 2001 ▶) *T*_min_ = 0.828, *T*_max_ = 0.892
18878 measured reflections
6655 independent reflections
5825 reflections with *I* > 2σ(*I*)
*R*_int_ = 0.018

#### Refinement

*R*[*F* ^2^ > 2σ(*F* ^2^)] = 0.029
*wR*(*F* ^2^) = 0.086
*S* = 1.00
6655 reflections
557 parameters
H atoms treated by a mixture of independent and constrained refinement
Δρ_max_ = 0.31 e Å^−3^
Δρ_min_ = −0.22 e Å^−3^

### 

Data collection: *APEX2* (Bruker, 2004 ▶); cell refinement: *SAINT-Plus* (Bruker, 2001 ▶); data reduction: *SAINT-Plus*; program(s) used to solve structure: *SHELXS97* (Sheldrick, 2008 ▶); program(s) used to refine structure: *SHELXL97* (Sheldrick, 2008 ▶); molecular graphics: *SHELXTL* (Sheldrick, 2008 ▶); software used to prepare material for publication: *SHELXTL*.

## Supplementary Material

Crystal structure: contains datablocks global, I. DOI: 10.1107/S1600536808035162/bi2307sup1.cif

Structure factors: contains datablocks I. DOI: 10.1107/S1600536808035162/bi2307Isup2.hkl

Additional supplementary materials:  crystallographic information; 3D view; checkCIF report

## Figures and Tables

**Table 1:** Hydrogen-bond geometry (Å, °)

*D*—H⋯*A*	*D*—H	H⋯*A*	*D*⋯*A*	*D*—H⋯*A*
N4—H4*A*⋯N6′^i^	0.86 (4)	2.68 (4)	3.463 (3)	154 (3)
N4—H4*A*⋯N5′^i^	0.86 (4)	2.69 (4)	3.514 (3)	163 (3)
N4′—H4*C*⋯N10^ii^	0.92 (3)	2.44 (2)	3.065 (3)	124.7 (18)
N4′—H4*D*⋯N13′^iii^	0.86 (3)	2.38 (3)	3.213 (3)	162 (2)

Symmetry codes: (i) 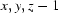; (ii) 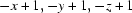; (iii) 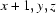.

**Fig. 1.**
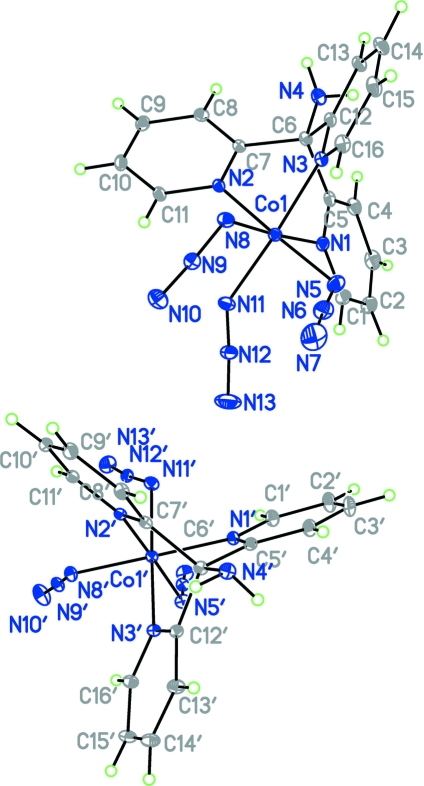
Two molecules in the asymmetric unit of the title compound with displacement ellipsoids shown at 30 % probability for non-H atoms.

## supplementary crystallographic information

### Comment

Pyridine-based ligands have been widely in materials science as ligands that can coordinate to transition-metal or rare-earth cations. The title compound is a Co^III^ triazide complex with the tridentate tris(2-pyridyl)methylamine ligand.

### Experimental

A mixture of hexaamminecobalt(III) nitrate (0.5 mmol), sodium azide (0.5 mmol), tris(2-pyridyl)methylamine (0.5 mmol), H_2_O (8 ml) and ethanol (8 ml) in a 25 ml Teflon-lined stainless steel autoclave was kept at 433 K for three days. Brown crystals were obtained after cooling to room temperature with a yield of 12 %. Elemental analysis calculated: C 42.92, H 3.13, N 40.69 %; found: C 42.88, H 3.06, N 40.55 %.

### Refinement

H atoms bound to C atoms were placed in calculated positions with C—H = 0.93 Å and refined as riding with *U*_iso_(H) = 1.2*U*_eq_(C). The H atoms of the amine groups were located in difference Fourier maps and refined without restraint.

### Crystal data

**Table:**

[Co(N_3_)_3_(C_16_H_14_N_4_)]	*Z* = 4
*M**_r_* = 447.33	*F*_000_ = 912
Triclinic, *P*1	*D*_x_ = 1.633 Mg m^−^^3^
Hall symbol: -P 1	Mo *K*α radiation λ = 0.71073 Å
*a* = 10.2063 (3) Å	Cell parameters from 6655 reflections
*b* = 13.6760 (6) Å	θ = 1.5–25.5º
*c* = 14.4735 (2) Å	µ = 0.98 mm^−^^1^
α = 68.150 (3)º	*T* = 295 (2) K
β = 77.114 (2)º	Block, brown
γ = 80.249 (2)º	0.20 × 0.14 × 0.12 mm
*V* = 1819.53 (10) Å^3^	

### Data collection

**Table:**

Bruker APEXII CCD diffractometer	6655 independent reflections
Radiation source: fine-focus sealed tube	5825 reflections with *I* > 2σ(*I*)
Monochromator: graphite	*R*_int_ = 0.018
*T* = 295(2) K	θ_max_ = 25.5º
φ and ω scans	θ_min_ = 1.5º
Absorption correction: multi-scan(SADABS; Bruker, 2001)	*h* = −12→12
*T*_min_ = 0.828, *T*_max_ = 0.892	*k* = −16→16
18878 measured reflections	*l* = −17→17

### Refinement

**Table:**

Refinement on *F*^2^	Secondary atom site location: difference Fourier map
Least-squares matrix: full	Hydrogen site location: inferred from neighbouring sites
*R*[*F*^2^ > 2σ(*F*^2^)] = 0.029	H atoms treated by a mixture of independent and constrained refinement
*wR*(*F*^2^) = 0.087	*w* = 1/[σ^2^(*F*_o_^2^) + (0.056*P*)^2^ + 0.548*P*] where *P* = (*F*_o_^2^ + 2*F*_c_^2^)/3
*S* = 1.00	(Δ/σ)_max_ = 0.033
6655 reflections	Δρ_max_ = 0.31 e Å^−^^3^
557 parameters	Δρ_min_ = −0.22 e Å^−^^3^
Primary atom site location: structure-invariant direct methods	Extinction correction: none

### Special details

**Table:**

Geometry. All e.s.d.'s (except the e.s.d. in the dihedral angle between two l.s. planes) are estimated using the full covariance matrix. The cell e.s.d.'s are taken into account individually in the estimation of e.s.d.'s in distances, angles and torsion angles; correlations between e.s.d.'s in cell parameters are only used when they are defined by crystal symmetry. An approximate (isotropic) treatment of cell e.s.d.'s is used for estimating e.s.d.'s involving l.s. planes.
Refinement. Refinement of *F*^2^ against ALL reflections. The weighted *R*-factor *wR* and goodness of fit *S* are based on *F*^2^, conventional *R*-factors *R* are based on *F*, with *F* set to zero for negative *F*^2^. The threshold expression of *F*^2^ > σ(*F*^2^) is used only for calculating *R*-factors(gt) *etc*. and is not relevant to the choice of reflections for refinement. *R*-factors based on *F*^2^ are statistically about twice as large as those based on *F*, and *R*- factors based on ALL data will be even larger.

### Fractional atomic coordinates and isotropic or equivalent isotropic displacement parameters (Å^2^)

**Table:**

	*x*	*y*	*z*	*U*_iso_*/*U*_eq_	
Co1'	0.08066 (2)	0.308970 (19)	0.810308 (17)	0.02644 (8)	
Co1	0.26516 (3)	0.20548 (2)	0.299922 (19)	0.03377 (9)	
C1	0.2380 (3)	0.0073 (2)	0.46611 (17)	0.0542 (6)	
H1	0.2814	0.0390	0.4962	0.065*	
C2	0.1936 (3)	−0.0916 (2)	0.5212 (2)	0.0685 (8)	
H2	0.2090	−0.1269	0.5871	0.082*	
C3	0.1266 (3)	−0.1370 (2)	0.4774 (2)	0.0690 (8)	
H3	0.0923	−0.2019	0.5146	0.083*	
C4	0.1102 (3)	−0.08588 (18)	0.37784 (19)	0.0530 (6)	
H4	0.0667	−0.1164	0.3467	0.064*	
C5	0.1600 (2)	0.01205 (16)	0.32530 (16)	0.0390 (5)	
C6	0.1434 (2)	0.07501 (15)	0.21526 (15)	0.0349 (4)	
C7	0.04845 (18)	0.17351 (15)	0.21651 (14)	0.0313 (4)	
C8	−0.0757 (2)	0.19521 (17)	0.18453 (15)	0.0404 (5)	
H8	−0.1040	0.1488	0.1613	0.048*	
C9	−0.1558 (2)	0.28565 (19)	0.18759 (17)	0.0471 (5)	
H9	−0.2392	0.3011	0.1668	0.057*	
C10	−0.1112 (2)	0.35281 (18)	0.22176 (17)	0.0471 (5)	
H10	−0.1637	0.4147	0.2240	0.056*	
C11	0.0128 (2)	0.32761 (16)	0.25290 (15)	0.0387 (5)	
H11	0.0426	0.3734	0.2762	0.046*	
C12	0.28077 (19)	0.11126 (15)	0.14989 (15)	0.0340 (4)	
C13	0.3317 (2)	0.08941 (18)	0.06194 (17)	0.0454 (5)	
H13	0.2849	0.0507	0.0413	0.054*	
C14	0.4534 (3)	0.12597 (19)	0.00521 (18)	0.0529 (6)	
H14	0.4884	0.1131	−0.0547	0.064*	
C15	0.5216 (2)	0.18087 (19)	0.03765 (19)	0.0537 (6)	
H15	0.6048	0.2039	0.0014	0.064*	
C16	0.4654 (2)	0.20184 (18)	0.12520 (17)	0.0453 (5)	
H16	0.5109	0.2407	0.1466	0.054*	
C1'	0.2190 (2)	0.11662 (17)	0.78384 (17)	0.0435 (5)	
H1'	0.1346	0.0918	0.8127	0.052*	
C2'	0.3226 (2)	0.05019 (19)	0.7545 (2)	0.0562 (6)	
H2'	0.3088	−0.0185	0.7635	0.067*	
C3'	0.4475 (2)	0.0874 (2)	0.7115 (2)	0.0571 (6)	
H3'	0.5192	0.0443	0.6904	0.069*	
C4'	0.4649 (2)	0.18962 (18)	0.70021 (18)	0.0453 (5)	
H4'	0.5487	0.2157	0.6717	0.054*	
C5'	0.35712 (18)	0.25276 (16)	0.73144 (14)	0.0317 (4)	
C6'	0.36740 (17)	0.36699 (16)	0.71976 (14)	0.0312 (4)	
C7'	0.26982 (18)	0.44037 (15)	0.65124 (13)	0.0292 (4)	
C8'	0.3112 (2)	0.52144 (17)	0.56277 (15)	0.0404 (5)	
H8'	0.4020	0.5330	0.5416	0.048*	
C9'	0.2168 (2)	0.58487 (18)	0.50625 (16)	0.0466 (5)	
H9'	0.2434	0.6394	0.4462	0.056*	
C10'	0.0833 (2)	0.56725 (17)	0.53900 (15)	0.0410 (5)	
H10'	0.0181	0.6102	0.5022	0.049*	
C11'	0.0474 (2)	0.48495 (16)	0.62732 (14)	0.0341 (4)	
H11'	−0.0430	0.4722	0.6490	0.041*	
C12'	0.32219 (18)	0.38099 (15)	0.82358 (14)	0.0306 (4)	
C13'	0.4033 (2)	0.41939 (18)	0.86443 (16)	0.0428 (5)	
H13'	0.4906	0.4344	0.8311	0.051*	
C14'	0.3538 (2)	0.4352 (2)	0.95506 (18)	0.0505 (6)	
H14'	0.4068	0.4620	0.9829	0.061*	
C15'	0.2253 (2)	0.41100 (18)	1.00387 (16)	0.0455 (5)	
H15'	0.1903	0.4211	1.0651	0.055*	
C16'	0.1495 (2)	0.37165 (16)	0.96077 (14)	0.0370 (4)	
H16'	0.0629	0.3545	0.9941	0.044*	
H4A	0.077 (3)	0.046 (3)	0.116 (3)	0.088 (12)*	
H4B	0.133 (3)	−0.039 (2)	0.180 (2)	0.053 (8)*	
H4C	0.510 (2)	0.463 (2)	0.6662 (17)	0.041 (6)*	
H4D	0.556 (3)	0.354 (2)	0.715 (2)	0.054 (8)*	
N1	0.21948 (17)	0.05876 (14)	0.36963 (13)	0.0397 (4)	
N2	0.09126 (15)	0.23921 (12)	0.25047 (11)	0.0311 (3)	
N3	0.34651 (16)	0.16761 (13)	0.18047 (12)	0.0350 (4)	
N4	0.0822 (2)	0.01220 (19)	0.17793 (18)	0.0477 (5)	
N5	0.4350 (2)	0.16394 (19)	0.34980 (17)	0.0590 (5)	
N6	0.4967 (2)	0.2294 (2)	0.35298 (19)	0.0679 (7)	
N7	0.5618 (3)	0.2892 (3)	0.3565 (3)	0.1143 (12)	
N8	0.31082 (19)	0.34925 (15)	0.22377 (14)	0.0442 (4)	
N9	0.30464 (17)	0.41107 (15)	0.26644 (14)	0.0437 (4)	
N10	0.2999 (2)	0.47478 (18)	0.30208 (18)	0.0608 (6)	
N11	0.1681 (2)	0.24176 (17)	0.41551 (14)	0.0497 (5)	
N12	0.2200 (2)	0.25807 (19)	0.47283 (15)	0.0576 (5)	
N13	0.2617 (3)	0.2758 (3)	0.5315 (2)	0.1140 (13)	
N1'	0.23509 (15)	0.21628 (12)	0.77228 (12)	0.0309 (3)	
N2'	0.13866 (14)	0.42244 (12)	0.68322 (11)	0.0276 (3)	
N3'	0.19653 (15)	0.35702 (12)	0.87168 (11)	0.0295 (3)	
N4'	0.50443 (17)	0.39349 (18)	0.67291 (15)	0.0407 (4)	
N5'	0.03456 (17)	0.19858 (14)	0.94046 (13)	0.0391 (4)	
N6'	−0.05808 (16)	0.14639 (13)	0.96236 (12)	0.0347 (4)	
N7'	−0.1435 (2)	0.09138 (17)	0.99093 (16)	0.0543 (5)	
N8'	−0.06488 (16)	0.40910 (13)	0.84197 (13)	0.0365 (4)	
N9'	−0.16651 (16)	0.38260 (14)	0.90016 (13)	0.0382 (4)	
N10'	−0.2668 (2)	0.36471 (19)	0.95508 (19)	0.0677 (7)	
N11'	−0.02658 (16)	0.26267 (15)	0.74183 (13)	0.0393 (4)	
N12'	−0.14505 (16)	0.25427 (13)	0.76507 (13)	0.0356 (4)	
N13'	−0.25777 (19)	0.23998 (19)	0.78241 (18)	0.0588 (6)	

### Atomic displacement parameters (Å^2^)

**Table:**

	*U*^11^	*U*^22^	*U*^33^	*U*^12^	*U*^13^	*U*^23^
Co1'	0.02280 (13)	0.02834 (15)	0.02953 (14)	−0.00705 (10)	−0.00219 (9)	−0.01100 (11)
Co1	0.03571 (15)	0.03377 (16)	0.03405 (15)	−0.00451 (11)	−0.00721 (11)	−0.01317 (12)
C1	0.0648 (15)	0.0494 (14)	0.0385 (12)	0.0027 (12)	−0.0097 (10)	−0.0072 (11)
C2	0.0843 (19)	0.0540 (16)	0.0415 (13)	0.0070 (15)	−0.0040 (13)	0.0027 (12)
C3	0.085 (2)	0.0351 (13)	0.0598 (16)	−0.0063 (13)	0.0103 (14)	0.0011 (12)
C4	0.0591 (14)	0.0327 (12)	0.0578 (14)	−0.0083 (10)	0.0051 (11)	−0.0124 (11)
C5	0.0373 (10)	0.0291 (10)	0.0439 (11)	−0.0003 (8)	0.0029 (8)	−0.0122 (9)
C6	0.0367 (10)	0.0284 (10)	0.0399 (10)	−0.0074 (8)	−0.0007 (8)	−0.0140 (8)
C7	0.0335 (9)	0.0297 (10)	0.0295 (9)	−0.0093 (8)	0.0014 (7)	−0.0102 (8)
C8	0.0386 (11)	0.0469 (13)	0.0390 (11)	−0.0096 (9)	−0.0050 (8)	−0.0175 (10)
C9	0.0364 (11)	0.0547 (14)	0.0476 (12)	0.0022 (10)	−0.0100 (9)	−0.0161 (11)
C10	0.0443 (12)	0.0408 (12)	0.0500 (12)	0.0088 (10)	−0.0074 (10)	−0.0150 (10)
C11	0.0452 (11)	0.0312 (10)	0.0398 (10)	−0.0024 (9)	−0.0027 (9)	−0.0157 (9)
C12	0.0346 (10)	0.0269 (10)	0.0379 (10)	−0.0005 (8)	−0.0024 (8)	−0.0117 (8)
C13	0.0518 (13)	0.0403 (12)	0.0457 (12)	−0.0014 (10)	−0.0021 (10)	−0.0219 (10)
C14	0.0590 (14)	0.0443 (13)	0.0473 (13)	−0.0017 (11)	0.0119 (11)	−0.0201 (11)
C15	0.0415 (12)	0.0458 (13)	0.0599 (14)	−0.0057 (10)	0.0147 (10)	−0.0155 (11)
C16	0.0351 (10)	0.0434 (12)	0.0553 (13)	−0.0087 (9)	−0.0005 (9)	−0.0169 (10)
C1'	0.0404 (11)	0.0338 (11)	0.0566 (13)	−0.0077 (9)	−0.0040 (9)	−0.0167 (10)
C2'	0.0507 (13)	0.0350 (12)	0.0838 (18)	−0.0008 (10)	−0.0073 (12)	−0.0257 (12)
C3'	0.0418 (12)	0.0473 (14)	0.0804 (17)	0.0102 (11)	−0.0055 (12)	−0.0294 (13)
C4'	0.0284 (10)	0.0500 (13)	0.0571 (13)	0.0000 (9)	−0.0034 (9)	−0.0221 (11)
C5'	0.0274 (9)	0.0366 (11)	0.0323 (9)	−0.0027 (8)	−0.0065 (7)	−0.0129 (8)
C6'	0.0229 (8)	0.0376 (11)	0.0338 (9)	−0.0092 (8)	0.0004 (7)	−0.0136 (8)
C7'	0.0295 (9)	0.0317 (10)	0.0296 (9)	−0.0069 (7)	−0.0002 (7)	−0.0155 (8)
C8'	0.0375 (10)	0.0395 (12)	0.0387 (11)	−0.0072 (9)	0.0058 (8)	−0.0131 (9)
C9'	0.0581 (13)	0.0367 (12)	0.0339 (10)	−0.0020 (10)	0.0019 (9)	−0.0064 (9)
C10'	0.0502 (12)	0.0374 (11)	0.0347 (10)	0.0052 (9)	−0.0120 (9)	−0.0132 (9)
C11'	0.0351 (10)	0.0372 (11)	0.0352 (10)	−0.0006 (8)	−0.0083 (8)	−0.0184 (9)
C12'	0.0294 (9)	0.0312 (10)	0.0317 (9)	−0.0070 (8)	−0.0065 (7)	−0.0092 (8)
C13'	0.0360 (10)	0.0532 (13)	0.0463 (12)	−0.0177 (10)	−0.0067 (9)	−0.0200 (10)
C14'	0.0542 (13)	0.0613 (15)	0.0495 (13)	−0.0175 (11)	−0.0167 (10)	−0.0251 (12)
C15'	0.0604 (14)	0.0508 (13)	0.0315 (10)	−0.0156 (11)	−0.0070 (9)	−0.0175 (10)
C16'	0.0419 (11)	0.0407 (11)	0.0286 (9)	−0.0135 (9)	−0.0006 (8)	−0.0115 (8)
N1	0.0420 (9)	0.0341 (9)	0.0351 (9)	0.0013 (7)	−0.0038 (7)	−0.0069 (7)
N2	0.0336 (8)	0.0272 (8)	0.0316 (8)	−0.0034 (6)	−0.0020 (6)	−0.0114 (7)
N3	0.0330 (8)	0.0321 (9)	0.0385 (9)	−0.0029 (7)	−0.0029 (7)	−0.0128 (7)
N4	0.0523 (12)	0.0377 (11)	0.0613 (14)	−0.0110 (10)	−0.0061 (10)	−0.0259 (11)
N5	0.0521 (12)	0.0670 (14)	0.0663 (13)	0.0004 (11)	−0.0281 (10)	−0.0250 (11)
N6	0.0386 (11)	0.100 (2)	0.0765 (16)	−0.0055 (12)	−0.0177 (10)	−0.0401 (15)
N7	0.0655 (17)	0.139 (3)	0.168 (3)	−0.0211 (19)	−0.043 (2)	−0.069 (3)
N8	0.0550 (11)	0.0400 (10)	0.0442 (10)	−0.0146 (8)	−0.0085 (8)	−0.0178 (9)
N9	0.0397 (10)	0.0428 (11)	0.0520 (11)	−0.0139 (8)	−0.0090 (8)	−0.0154 (9)
N10	0.0627 (13)	0.0567 (13)	0.0775 (15)	−0.0207 (11)	−0.0087 (11)	−0.0352 (12)
N11	0.0538 (11)	0.0620 (13)	0.0423 (10)	−0.0063 (9)	−0.0073 (8)	−0.0286 (9)
N12	0.0605 (12)	0.0751 (15)	0.0439 (11)	0.0040 (11)	−0.0146 (10)	−0.0297 (11)
N13	0.107 (2)	0.188 (4)	0.086 (2)	0.003 (2)	−0.0369 (18)	−0.088 (2)
N1'	0.0264 (7)	0.0304 (8)	0.0365 (8)	−0.0039 (6)	−0.0047 (6)	−0.0123 (7)
N2'	0.0273 (7)	0.0304 (8)	0.0282 (7)	−0.0045 (6)	−0.0034 (6)	−0.0138 (6)
N3'	0.0302 (8)	0.0315 (8)	0.0268 (7)	−0.0087 (6)	−0.0034 (6)	−0.0086 (6)
N4'	0.0257 (8)	0.0520 (12)	0.0453 (10)	−0.0134 (8)	0.0011 (7)	−0.0177 (9)
N5'	0.0377 (9)	0.0398 (10)	0.0368 (9)	−0.0163 (8)	−0.0025 (7)	−0.0069 (8)
N6'	0.0352 (9)	0.0331 (9)	0.0335 (8)	−0.0065 (8)	−0.0016 (7)	−0.0102 (7)
N7'	0.0510 (11)	0.0525 (12)	0.0569 (12)	−0.0245 (10)	−0.0030 (9)	−0.0115 (10)
N8'	0.0291 (8)	0.0353 (9)	0.0429 (9)	−0.0045 (7)	0.0024 (7)	−0.0152 (8)
N9'	0.0342 (9)	0.0373 (10)	0.0487 (10)	−0.0061 (7)	−0.0017 (8)	−0.0234 (8)
N10'	0.0462 (11)	0.0675 (15)	0.0875 (16)	−0.0213 (11)	0.0271 (11)	−0.0402 (13)
N11'	0.0323 (9)	0.0476 (11)	0.0482 (10)	−0.0111 (8)	−0.0065 (7)	−0.0255 (8)
N12'	0.0356 (9)	0.0318 (9)	0.0439 (9)	−0.0032 (7)	−0.0140 (7)	−0.0145 (7)
N13'	0.0329 (10)	0.0718 (15)	0.0788 (14)	−0.0106 (9)	−0.0164 (9)	−0.0281 (12)

### Geometric parameters (Å, °)

**Table:**

Co1'—N11'	1.9408 (17)	C1'—H1'	0.930
Co1'—N8'	1.9407 (16)	C2'—C3'	1.379 (4)
Co1'—N5'	1.9436 (16)	C2'—H2'	0.930
Co1'—N3'	1.9456 (16)	C3'—C4'	1.383 (3)
Co1'—N2'	1.9603 (15)	C3'—H3'	0.930
Co1'—N1'	1.9621 (15)	C4'—C5'	1.381 (3)
Co1—N11	1.9335 (18)	C4'—H4'	0.930
Co1—N8	1.9406 (18)	C5'—N1'	1.346 (2)
Co1—N5	1.941 (2)	C5'—C6'	1.527 (3)
Co1—N3	1.9563 (17)	C6'—N4'	1.454 (2)
Co1—N1	1.9651 (18)	C6'—C7'	1.531 (3)
Co1—N2	1.9796 (16)	C6'—C12'	1.544 (3)
C1—N1	1.350 (3)	C7'—N2'	1.348 (2)
C1—C2	1.384 (4)	C7'—C8'	1.381 (3)
C1—H1	0.930	C8'—C9'	1.375 (3)
C2—C3	1.374 (4)	C8'—H8'	0.930
C2—H2	0.930	C9'—C10'	1.370 (3)
C3—C4	1.382 (4)	C9'—H9'	0.930
C3—H3	0.930	C10'—C11'	1.377 (3)
C4—C5	1.390 (3)	C10'—H10'	0.930
C4—H4	0.930	C11'—N2'	1.344 (2)
C5—N1	1.341 (3)	C11'—H11'	0.930
C5—C6	1.535 (3)	C12'—N3'	1.346 (2)
C6—N4	1.448 (3)	C12'—C13'	1.381 (3)
C6—C7	1.524 (3)	C13'—C14'	1.379 (3)
C6—C12	1.550 (3)	C13'—H13'	0.930
C7—N2	1.341 (3)	C14'—C15'	1.374 (3)
C7—C8	1.392 (3)	C14'—H14'	0.930
C8—C9	1.372 (3)	C15'—C16'	1.372 (3)
C8—H8	0.930	C15'—H15'	0.930
C9—C10	1.370 (3)	C16'—N3'	1.348 (2)
C9—H9	0.930	C16'—H16'	0.930
C10—C11	1.384 (3)	N4—H4A	0.86 (4)
C10—H10	0.930	N4—H4B	0.79 (3)
C11—N2	1.340 (2)	N5—N6	1.198 (3)
C11—H11	0.930	N6—N7	1.159 (4)
C12—N3	1.342 (3)	N8—N9	1.206 (3)
C12—C13	1.384 (3)	N9—N10	1.156 (3)
C13—C14	1.385 (3)	N11—N12	1.180 (3)
C13—H13	0.930	N12—N13	1.145 (3)
C14—C15	1.361 (4)	N4'—H4C	0.92 (3)
C14—H14	0.930	N4'—H4D	0.86 (3)
C15—C16	1.381 (3)	N5'—N6'	1.196 (2)
C15—H15	0.930	N6'—N7'	1.152 (2)
C16—N3	1.346 (3)	N8'—N9'	1.195 (2)
C16—H16	0.930	N9'—N10'	1.147 (2)
C1'—N1'	1.344 (3)	N11'—N12'	1.194 (2)
C1'—C2'	1.373 (3)	N12'—N13'	1.156 (2)
			
N11'—Co1'—N8'	92.84 (7)	C3'—C2'—C1'	118.7 (2)
N11'—Co1'—N5'	94.69 (7)	C3'—C2'—H2'	120.7
N8'—Co1'—N5'	93.10 (7)	C1'—C2'—H2'	120.7
N11'—Co1'—N3'	176.55 (7)	C2'—C3'—C4'	119.2 (2)
N8'—Co1'—N3'	89.23 (7)	C2'—C3'—H3'	120.4
N5'—Co1'—N3'	87.95 (7)	C4'—C3'—H3'	120.4
N11'—Co1'—N2'	89.69 (7)	C5'—C4'—C3'	119.6 (2)
N8'—Co1'—N2'	87.79 (7)	C5'—C4'—H4'	120.2
N5'—Co1'—N2'	175.48 (7)	C3'—C4'—H4'	120.2
N3'—Co1'—N2'	87.63 (6)	N1'—C5'—C4'	120.82 (19)
N11'—Co1'—N1'	88.91 (7)	N1'—C5'—C6'	116.36 (15)
N8'—Co1'—N1'	175.94 (7)	C4'—C5'—C6'	122.80 (17)
N5'—Co1'—N1'	90.40 (7)	N4'—C6'—C5'	108.89 (16)
N3'—Co1'—N1'	88.84 (7)	N4'—C6'—C7'	109.04 (16)
N2'—Co1'—N1'	88.57 (6)	C5'—C6'—C7'	108.97 (15)
N11—Co1—N8	92.79 (9)	N4'—C6'—C12'	113.23 (16)
N11—Co1—N5	94.33 (9)	C5'—C6'—C12'	109.64 (15)
N8—Co1—N5	92.47 (9)	C7'—C6'—C12'	106.98 (15)
N11—Co1—N3	174.49 (8)	N2'—C7'—C8'	121.22 (17)
N8—Co1—N3	88.90 (7)	N2'—C7'—C6'	115.82 (15)
N5—Co1—N3	90.83 (8)	C8'—C7'—C6'	122.96 (16)
N11—Co1—N1	90.12 (8)	C9'—C8'—C7'	119.35 (19)
N8—Co1—N1	176.71 (8)	C9'—C8'—H8'	120.3
N5—Co1—N1	88.82 (9)	C7'—C8'—H8'	120.3
N3—Co1—N1	88.06 (7)	C10'—C9'—C8'	119.51 (19)
N11—Co1—N2	86.79 (7)	C10'—C9'—H9'	120.2
N8—Co1—N2	90.55 (7)	C8'—C9'—H9'	120.2
N5—Co1—N2	176.72 (8)	C9'—C10'—C11'	118.90 (19)
N3—Co1—N2	87.95 (7)	C9'—C10'—H10'	120.5
N1—Co1—N2	88.10 (7)	C11'—C10'—H10'	120.5
N1—C1—C2	121.4 (3)	N2'—C11'—C10'	122.12 (18)
N1—C1—H1	119.3	N2'—C11'—H11'	118.9
C2—C1—H1	119.3	C10'—C11'—H11'	118.9
C3—C2—C1	119.1 (2)	N3'—C12'—C13'	121.07 (18)
C3—C2—H2	120.4	N3'—C12'—C6'	116.41 (16)
C1—C2—H2	120.4	C13'—C12'—C6'	122.49 (16)
C2—C3—C4	119.8 (2)	C14'—C13'—C12'	119.39 (19)
C2—C3—H3	120.1	C14'—C13'—H13'	120.3
C4—C3—H3	120.1	C12'—C13'—H13'	120.3
C3—C4—C5	118.5 (3)	C15'—C14'—C13'	119.4 (2)
C3—C4—H4	120.7	C15'—C14'—H14'	120.3
C5—C4—H4	120.7	C13'—C14'—H14'	120.3
N1—C5—C4	121.7 (2)	C16'—C15'—C14'	118.86 (19)
N1—C5—C6	116.48 (17)	C16'—C15'—H15'	120.6
C4—C5—C6	121.8 (2)	C14'—C15'—H15'	120.6
N4—C6—C7	109.19 (18)	N3'—C16'—C15'	122.15 (18)
N4—C6—C5	109.42 (18)	N3'—C16'—H16'	118.9
C7—C6—C5	106.55 (16)	C15'—C16'—H16'	118.9
N4—C6—C12	113.13 (17)	C5—N1—C1	119.4 (2)
C7—C6—C12	108.01 (15)	C5—N1—Co1	120.66 (14)
C5—C6—C12	110.32 (16)	C1—N1—Co1	119.71 (17)
N2—C7—C8	121.29 (18)	C11—N2—C7	118.97 (17)
N2—C7—C6	116.02 (17)	C11—N2—Co1	119.98 (14)
C8—C7—C6	122.69 (18)	C7—N2—Co1	121.03 (13)
C9—C8—C7	119.5 (2)	C16—N3—C12	119.15 (18)
C9—C8—H8	120.3	C16—N3—Co1	120.69 (15)
C7—C8—H8	120.3	C12—N3—Co1	120.07 (12)
C10—C9—C8	119.0 (2)	C6—N4—H4A	109 (2)
C10—C9—H9	120.5	C6—N4—H4B	107 (2)
C8—C9—H9	120.5	H4A—N4—H4B	107 (3)
C9—C10—C11	119.4 (2)	N6—N5—Co1	120.19 (19)
C9—C10—H10	120.3	N7—N6—N5	176.7 (3)
C11—C10—H10	120.3	N9—N8—Co1	120.37 (15)
N2—C11—C10	121.9 (2)	N10—N9—N8	176.1 (2)
N2—C11—H11	119.0	N12—N11—Co1	124.36 (17)
C10—C11—H11	119.0	N13—N12—N11	175.3 (3)
N3—C12—C13	121.27 (18)	C5'—N1'—C1'	119.33 (17)
N3—C12—C6	117.20 (16)	C5'—N1'—Co1'	120.43 (13)
C13—C12—C6	121.50 (19)	C1'—N1'—Co1'	120.21 (13)
C14—C13—C12	119.0 (2)	C11'—N2'—C7'	118.88 (16)
C14—C13—H13	120.5	C11'—N2'—Co1'	120.25 (12)
C12—C13—H13	120.5	C7'—N2'—Co1'	120.86 (12)
C15—C14—C13	119.6 (2)	C12'—N3'—C16'	119.08 (16)
C15—C14—H14	120.2	C12'—N3'—Co1'	120.54 (12)
C13—C14—H14	120.2	C16'—N3'—Co1'	120.30 (13)
C14—C15—C16	119.1 (2)	C6'—N4'—H4C	108.3 (14)
C14—C15—H15	120.5	C6'—N4'—H4D	106.8 (17)
C16—C15—H15	120.5	H4C—N4'—H4D	107 (2)
N3—C16—C15	121.9 (2)	N6'—N5'—Co1'	124.28 (14)
N3—C16—H16	119.1	N7'—N6'—N5'	174.3 (2)
C15—C16—H16	119.1	N9'—N8'—Co1'	123.02 (14)
N1'—C1'—C2'	122.4 (2)	N10'—N9'—N8'	175.1 (2)
N1'—C1'—H1'	118.8	N12'—N11'—Co1'	126.01 (14)
C2'—C1'—H1'	118.8	N13'—N12'—N11'	173.6 (2)
			
N1—C1—C2—C3	−1.5 (4)	N8—Co1—N2—C11	−46.96 (15)
C1—C2—C3—C4	3.1 (4)	N3—Co1—N2—C11	−135.84 (15)
C2—C3—C4—C5	−1.4 (4)	N1—Co1—N2—C11	136.03 (15)
C3—C4—C5—N1	−2.0 (3)	N11—Co1—N2—C7	−132.58 (15)
C3—C4—C5—C6	−178.7 (2)	N8—Co1—N2—C7	134.66 (14)
N1—C5—C6—N4	178.02 (17)	N3—Co1—N2—C7	45.78 (14)
C4—C5—C6—N4	−5.1 (3)	N1—Co1—N2—C7	−42.35 (14)
N1—C5—C6—C7	−64.1 (2)	C13—C12—N3—C16	−1.1 (3)
C4—C5—C6—C7	112.9 (2)	C6—C12—N3—C16	−179.17 (18)
N1—C5—C6—C12	52.9 (2)	C13—C12—N3—Co1	175.64 (15)
C4—C5—C6—C12	−130.1 (2)	C6—C12—N3—Co1	−2.4 (2)
N4—C6—C7—N2	179.37 (17)	C15—C16—N3—C12	0.2 (3)
C5—C6—C7—N2	61.3 (2)	C15—C16—N3—Co1	−176.57 (17)
C12—C6—C7—N2	−57.2 (2)	N8—Co1—N3—C12	−133.04 (15)
N4—C6—C7—C8	−0.6 (3)	N5—Co1—N3—C12	134.50 (16)
C5—C6—C7—C8	−118.7 (2)	N1—Co1—N3—C12	45.72 (15)
C12—C6—C7—C8	122.74 (19)	N2—Co1—N3—C12	−42.45 (15)
N2—C7—C8—C9	0.1 (3)	N8—Co1—N3—C16	43.67 (17)
C6—C7—C8—C9	−179.86 (18)	N5—Co1—N3—C16	−48.78 (17)
C7—C8—C9—C10	0.3 (3)	N1—Co1—N3—C16	−137.57 (16)
C8—C9—C10—C11	−0.4 (3)	N2—Co1—N3—C16	134.27 (16)
C9—C10—C11—N2	0.1 (3)	N11—Co1—N5—N6	−70.1 (2)
N4—C6—C12—N3	−178.29 (19)	N8—Co1—N5—N6	22.9 (2)
C7—C6—C12—N3	60.7 (2)	N3—Co1—N5—N6	111.8 (2)
C5—C6—C12—N3	−55.3 (2)	N1—Co1—N5—N6	−160.2 (2)
N4—C6—C12—C13	3.7 (3)	N11—Co1—N8—N9	18.42 (18)
C7—C6—C12—C13	−117.3 (2)	N5—Co1—N8—N9	−76.04 (19)
C5—C6—C12—C13	126.6 (2)	N3—Co1—N8—N9	−166.82 (18)
N3—C12—C13—C14	0.5 (3)	N2—Co1—N8—N9	105.24 (18)
C6—C12—C13—C14	178.5 (2)	N8—Co1—N11—N12	−74.7 (2)
C12—C13—C14—C15	1.1 (3)	N5—Co1—N11—N12	18.0 (2)
C13—C14—C15—C16	−2.0 (4)	N1—Co1—N11—N12	106.8 (2)
C14—C15—C16—N3	1.4 (4)	N2—Co1—N11—N12	−165.1 (2)
N1'—C1'—C2'—C3'	−0.1 (4)	C2'—C1'—N1'—C5'	−0.6 (3)
C1'—C2'—C3'—C4'	0.6 (4)	C2'—C1'—N1'—Co1'	177.29 (19)
C2'—C3'—C4'—C5'	−0.4 (4)	C4'—C5'—N1'—C1'	0.9 (3)
C3'—C4'—C5'—N1'	−0.4 (3)	C6'—C5'—N1'—C1'	179.44 (18)
C3'—C4'—C5'—C6'	−178.8 (2)	C4'—C5'—N1'—Co1'	−177.02 (15)
N1'—C5'—C6'—N4'	−178.84 (16)	C6'—C5'—N1'—Co1'	1.5 (2)
C4'—C5'—C6'—N4'	−0.3 (3)	N11'—Co1'—N1'—C1'	−45.45 (16)
N1'—C5'—C6'—C7'	−60.0 (2)	N5'—Co1'—N1'—C1'	49.24 (17)
C4'—C5'—C6'—C7'	118.5 (2)	N3'—Co1'—N1'—C1'	137.18 (16)
N1'—C5'—C6'—C12'	56.8 (2)	N2'—Co1'—N1'—C1'	−135.16 (16)
C4'—C5'—C6'—C12'	−124.7 (2)	N11'—Co1'—N1'—C5'	132.45 (15)
N4'—C6'—C7'—N2'	177.58 (16)	N5'—Co1'—N1'—C5'	−132.86 (15)
C5'—C6'—C7'—N2'	58.9 (2)	N3'—Co1'—N1'—C5'	−44.92 (14)
C12'—C6'—C7'—N2'	−59.6 (2)	N2'—Co1'—N1'—C5'	42.74 (14)
N4'—C6'—C7'—C8'	−3.4 (3)	C10'—C11'—N2'—C7'	−0.6 (3)
C5'—C6'—C7'—C8'	−122.16 (19)	C10'—C11'—N2'—Co1'	178.25 (15)
C12'—C6'—C7'—C8'	119.37 (19)	C8'—C7'—N2'—C11'	0.1 (3)
N2'—C7'—C8'—C9'	−0.1 (3)	C6'—C7'—N2'—C11'	179.11 (16)
C6'—C7'—C8'—C9'	−179.01 (19)	C8'—C7'—N2'—Co1'	−178.76 (15)
C7'—C8'—C9'—C10'	0.5 (3)	C6'—C7'—N2'—Co1'	0.2 (2)
C8'—C9'—C10'—C11'	−1.0 (3)	N8'—Co1'—N2'—C11'	−44.40 (15)
C9'—C10'—C11'—N2'	1.1 (3)	N11'—Co1'—N2'—C11'	48.46 (15)
N4'—C6'—C12'—N3'	−179.50 (17)	N3'—Co1'—N2'—C11'	−133.72 (15)
C5'—C6'—C12'—N3'	−57.7 (2)	N1'—Co1'—N2'—C11'	137.38 (14)
C7'—C6'—C12'—N3'	60.4 (2)	N8'—Co1'—N2'—C7'	134.45 (14)
N4'—C6'—C12'—C13'	2.7 (3)	N11'—Co1'—N2'—C7'	−132.69 (14)
C5'—C6'—C12'—C13'	124.5 (2)	N3'—Co1'—N2'—C7'	45.13 (14)
C7'—C6'—C12'—C13'	−117.5 (2)	N1'—Co1'—N2'—C7'	−43.77 (14)
N3'—C12'—C13'—C14'	−1.2 (3)	C13'—C12'—N3'—C16'	0.5 (3)
C6'—C12'—C13'—C14'	176.6 (2)	C6'—C12'—N3'—C16'	−177.43 (17)
C12'—C13'—C14'—C15'	1.0 (4)	C13'—C12'—N3'—Co1'	177.26 (16)
C13'—C14'—C15'—C16'	0.0 (4)	C6'—C12'—N3'—Co1'	−0.6 (2)
C14'—C15'—C16'—N3'	−0.7 (3)	C15'—C16'—N3'—C12'	0.5 (3)
C4—C5—N1—C1	3.5 (3)	C15'—C16'—N3'—Co1'	−176.31 (16)
C6—C5—N1—C1	−179.55 (18)	N8'—Co1'—N3'—C12'	−132.36 (15)
C4—C5—N1—Co1	−171.08 (16)	N5'—Co1'—N3'—C12'	134.51 (15)
C6—C5—N1—Co1	5.8 (2)	N2'—Co1'—N3'—C12'	−44.53 (14)
C2—C1—N1—C5	−1.8 (3)	N1'—Co1'—N3'—C12'	44.08 (15)
C2—C1—N1—Co1	172.90 (19)	N8'—Co1'—N3'—C16'	44.41 (15)
N11—Co1—N1—C5	126.64 (16)	N5'—Co1'—N3'—C16'	−48.72 (15)
N5—Co1—N1—C5	−139.03 (16)	N2'—Co1'—N3'—C16'	132.23 (15)
N3—Co1—N1—C5	−48.16 (15)	N1'—Co1'—N3'—C16'	−139.16 (15)
N2—Co1—N1—C5	39.85 (15)	N8'—Co1'—N5'—N6'	76.66 (18)
N11—Co1—N1—C1	−47.94 (17)	N11'—Co1'—N5'—N6'	−16.46 (18)
N5—Co1—N1—C1	46.39 (18)	N3'—Co1'—N5'—N6'	165.77 (18)
N3—Co1—N1—C1	137.25 (17)	N1'—Co1'—N5'—N6'	−105.40 (18)
N2—Co1—N1—C1	−134.73 (17)	N11'—Co1'—N8'—N9'	67.91 (18)
C10—C11—N2—C7	0.3 (3)	N5'—Co1'—N8'—N9'	−26.95 (18)
C10—C11—N2—Co1	−178.16 (16)	N3'—Co1'—N8'—N9'	−114.85 (18)
C8—C7—N2—C11	−0.4 (3)	N2'—Co1'—N8'—N9'	157.49 (18)
C6—C7—N2—C11	179.59 (16)	N8'—Co1'—N11'—N12'	−35.41 (19)
C8—C7—N2—Co1	178.01 (14)	N5'—Co1'—N11'—N12'	57.94 (19)
C6—C7—N2—Co1	−2.0 (2)	N2'—Co1'—N11'—N12'	−123.19 (18)
N11—Co1—N2—C11	45.80 (15)	N1'—Co1'—N11'—N12'	148.24 (19)

### Hydrogen-bond geometry (Å, °)

**Table:**

*D*—H···*A*	*D*—H	H···*A*	*D*···*A*	*D*—H···*A*
N4—H4A···N6'^i^	0.86 (4)	2.68 (4)	3.463 (3)	154 (3)
N4—H4A···N5'^i^	0.86 (4)	2.69 (4)	3.514 (3)	163 (3)
N4'—H4C···N10^ii^	0.92 (3)	2.44 (2)	3.065 (3)	124.7 (18)
N4'—H4D···N13'^iii^	0.86 (3)	2.38 (3)	3.213 (3)	162 (2)

Symmetry codes: (i) *x*, *y*, *z*−1; (ii) −*x*+1, −*y*+1, −*z*+1; (iii) *x*+1, *y*, *z*.
